# Neurocutaneous melanosis presenting with hydrocephalus and malignant transformation: case-based update

**DOI:** 10.1007/s00381-018-3851-5

**Published:** 2018-06-12

**Authors:** F. Sharouf, M. Zaben, A. Lammie, P. Leach, M. I. Bhatti

**Affiliations:** 1 University Hospital of Wales, Department of Neurosurgery, Cardiff University, Heath Park, Cardiff, CF14 4XW UK; 2 University Hospital of Wales, Cardiff, UK

**Keywords:** Neurocutaneous melanosis, Hydrocephalus, Melanocytic nevi, Leptomeningeal melanosis

## Abstract

**Introduction:**

Neurocutaneous melanosis (NCM) is a sporadic condition characterised by congenital melanocytic nevi and melanocytic thickening of the leptomeninges. It is believed to result from congenital dysplasia of melanin-producing cells within the skin and leptomeninges. The management of cutaneous manifestations remains controversial; for neurological manifestations, outcome remains poor even with the use of radiotherapy and chemotherapy.

**Patients and methods:**

We describe the case of a 5-month-old boy who presented with giant congenital melanocytic nevus and hydrocephalus. MR imaging and CSF immunohistochemistry confirmed leptomeningeal melanosis. We discuss the diagnosis, treatment and prognosis of this rare disorder in the light of recent published literature.

**Results:**

Patient required placement of right-sided ventriculoperitoneal shunt to control hydrocephalus. The patient tolerated the procedure well and was discharged home with normal neurological function. A presumptive diagnosis of NCM was made based on the MR characteristics, CSF cytology and clinical presentation. He received trametinib, a MAPK/Erk kinase inhibitor for 7 months. At 30 months of age, he developed left-sided weakness and status epilepticus requiring paediatric intensive care unit admission and ventilator support. The patient eventually succumbed to malignant transformation of leptomeningeal disease.

**Conclusion:**

Cutaneous manifestations of NCM are usually congenital, and neurological manifestations develop early in life. Patients with large or multiple congenital nevi should therefore be investigated early to facilitate treatment. MR imaging is the investigation of choice which can further assist in performing biopsy. Symptomatic NCM is refractory to radiotherapy and chemotherapy and has a poor prognosis. A multidisciplinary approach is necessary in the management of NCM patients.

## Introduction

Neurocutaneous melanosis (NCM) is a rare syndrome characterised by congenital melanocytic nevi and melanocytic thickening of the leptomeninges [32]. Although mostly sporadic, a few familial cases of NCM have been reported [22]. In most cases, NCM presents with symptoms of raised intracranial pressure [1]. NCM is believed to result from congenital dysplasia of melanin-producing cells within the skin and leptomeninges [20].

Two thirds of patients with NCM have a giant congenital melanocytic nevus, and the remaining third have multiple small lesions [19]. Nevi are usually present at birth, but more may develop later in life. Almost all nevi have a lumbosacral (bathing trunk) distribution [10].

In the majority of cases, NCM exhibits symptoms of raised intracranial pressure within the first 2 years of life [20]. As in the case reported here, most cases present with symptoms and signs of increased intracranial pressure including irritability, lethargy, recurrent vomiting, increased head circumference, bulging anterior fontanelle and photophobia [1]. Hydrocephalus develops in two thirds of patients.

## Historical background

Although first described by Rokitanski in 1861 [31], the term neurocutaneous melanosis was coined by van Bogaert in 1948 [38]. The initial diagnostic criteria of NCM included large or numerous pigmented nevi without malignant transformation [12], which was later revised to include malignant transformation and distant metastasis [10]. Since its first description, 100 or so cases have been described in the English literature [28].

NCM is a sporadic syndrome with few reported familial cases [9, 13]. Animal models of NCM have been developed. Transgenic mice overexpressing hepatocyte growth factor/scatter factor (HGF/SF) demonstrate extensive pigmented nevi in both skin and leptomeninges of the central nervous system, thus resembling human NCM. HGF/SF are growth factors that control the proliferation of neural crest melanocytes during embryogenesis [37]. Dysregulation of these growth factors may explain associated cystic malformations of the posterior fossa such as the Dandy–Walker complex (DWC) [37].

Oncogenic missense mutations (affecting the NRAS gene) have been identified in affected neural and cutaneous tissue in NCM patients. However, these mutations were not found in unaffected tissues and blood. The mutations are thought to be the result of somatic mosaicism, which occurs in a progenitor cell in the developing neural crest or neuroectoderm [21]. This suggests that these mutations would be lethal if they occurred in germ line cells [21].

The resultant phenotype is dependent upon the type of mutation, affected cells and timing [15]. NRAS mutations have only been found in benign melanocytic nevi. This indicates that they are of themselves insufficient for malignant transformation to occur. Given that malignant transformation is an indicator of poor prognosis in NCM (as discussed in prognosis and outcomes), a better understanding of molecular genetic pathogenesis is required [39]. NRAS mutations could represent a potential therapeutic target for NCM [27]. NRAS melanomas are thought to proliferate through the MAPK pathway which could be inhibited by MEK inhibitors. Trametinib for example, a MEK inhibitor, has been approved by The Food and Drug Administration (FDA) for the treatment of certain NRAS-mutated melanomas [18].

The histopathological patterns of NCM cutaneous lesions are indistinguishable from those seen in congenital melanocytic nevi without CNS involvement. Nevus cells spread into the dermis and encircle nerves and blood vessels [19, 26]. Leptomeningeal melanosis is most evident in areas of physiological melanin distribution such as the base of the brain, the ventral surface of the pons, cerebral peduncles, the medulla and cerebellum [10]. Several features have been identified to distinguish meningeal melanosis from melanoma which can develop in about half of the cases [30]. Necrosis, invasion of basal lamina and cell atypia can distinguish melanoma from melanosis. Although the prognostic significance of this distinction is unclear [20], CSF cytology is used to investigate malignancy but its sensitivity is reported to be around 40% [20].

## Clinical presentation

A large congenital melanocytic nevus with bathing trunk distribution is observed in two thirds of patients with NCM [19]. In the remaining third of patients, multiple smaller melanocytic nevi without a single large lesion are found. Nevi are dark pigmented lesions circumscribed with irregular borders that can be raised or flat. They are usually present at birth, although new nevi can develop later in life [6].

Most patients exhibit neurological symptoms by 2 years of age; however, some cases may present during the second or third decade of life [20]. Two thirds of patients will present with neurological signs and symptoms due to increased intracranial pressure such as irritability, lethargy, headache, vomiting, photophobia, papilledema bulging anterior fontanel and enlarging head circumference [1], as in our exemplary case. Seizures, aphasia and motor or cranial nerve palsies may develop in patients with intracranial melanocytic tumours (see Table 1) [19]. Patients who present late in life may have spinal involvement which may result in myelopathy, radiculopathy and bowel or bladder dysfunction [19].

**Table 1:** Literature summary of NCM (DLE, diffuse leptomeningeal enhancement; ND, not done; M, male; F, female; EVD, external ventricular drain; VP shunt, ventriculoperitoneal shunt; DWC, Dandy–Walker complex)

Authors	Age of presentation	Gender	Symptoms	MRI findings	Histology	Treatment
Peters et al.2000 [29]	3 weeks	M	Seizure, hydrocephalus	Hydrocephalus, small hyperintense lesions in temporal lobes on T1W, enhancement lumbosacral region	ND	VP shunt, shunt from cisterna magna to peritoneum
Mena-Cedillos et al.2002 [26]	5 years	M	Hydrocephalus	ND	(Autopsy) melanoma cells	VP shunt
Shinno et al.2003 [33]	35 years	M	Hydrocephalus	DLE brain and spine	(Autopsy) melanoma cells	VP shunt, 6 courses of chemotherapy
Hsueh et al.2004 [16]	46 days	M	Hydrocephalus	Enhancement on T1-weighted images over the cerebellum, bilateral medial temporal lobes and ventral pons	Biopsy	EVD
Arai et al.2004 [3]	31 week gestation	M	Cystic mass in the occipital region and loss of occipital bone on US	DWC associated with an occipital meningocele	ND	VP and cystoperitoneal shunt, resection of skin lesion
de Andrade et al.2004 [8]	20 years	F	Seizure	DLE pons, the forebrain, and the two temporal lobes? DWC	ND	Resection of skin lesion
Di Rocco et al.2004 [11]	2 years and 7 months	F	Seizure	DLE brain, cystic lesions, DWC	Biopsy	Resection of tumour
Acosta et al.2005 [1]	5 months	F	Hydrocephalus	Melanocyte accumulation within the hippocampi, medulla and cerebellum	Biopsy	VP shunt
Iwabuchi et al.2005 [17]	26 week gestation	M	Hydrocephalus	ND	ND	VP shunt
McClelland et al.2007 [25]	1 year	M	Hydrocephalus	DLE brain and spine, DWC, hyperdense area of the left temporal lobe consistent with melanocyte pigmentation	Biopsy	VP shunt, posterior laminectomy from C-1 to C-3 and small midline suboccipital craniotomy
Pavlidou et al.2008 [28]	6 months	M	Seizure	ND	Biopsy	Conservative
	9 months	F	Seizure	Accumulation of melanocytic cells close to the amygdala and the cerebellum	Biopsy	Conservative
	6 years	M	Seizure, hydrocephalus, left hemiplegia, 7th nerve palsy	Marked hydrocephalus	Biopsy	VP shunt, chemotherapy and radiation
Marnet et al.2009 [24]	14 years	M	Hydrocephalus	Hydrocephalus, DWC	Biopsy	Cystoperitoneal shunt, VP shunt, chemotherapy
Cho et al.2011 [6]	2 months	F	Hydrocephalus, seizure	Hydrocephalus, DWC	ND	VP shunt
Swar et al.2013 [35]	3 months	M	Hydrocephalus	ND	ND	VP shunt
Yamazaki et al.2013 [40]	9 years	F	Headache, hydrocephalus, seizure	High-intensity areas on the right sulci, hydrocephalus	Autopsy	VP shunt, chemotherapy
Yoo et al. 2014 [41]	2 years and 4 months	F	Hydrocephalus, disseminated melanotic tumour via VP shunt	Enhancing extra-axial mass along the cerebrospinal fluid (CSF) spaces	Biopsy	VP shunt, chemotherapy
Sung et al.2014 [34]	2 years	M	Hydrocephalus, seizure, motor weakness	DLE brain and spine, cyst in the posterior fossa, DWC	Biopsy	Decompression of tumour

We reviewed the literature by searching PubMed using the terms “neurocutaneous melanosis” and “hydrocephalus.” We included only publications in English dated 01/01/2000 to present as illustrated in Table 1.

Neurocutaneous melanosis can be linked to other neurocutaneous disorders, such as neurofibromatosis type 1 and Sturge–Weber syndrome [10].

The association of Dandy-Walker Syndrome and NCM is a rare complex, and to our knowledge only 24 cases have been reported up do date [5, 11, 20, 26].

DWC may be due to the leptomeningeal anomalies of NCM, which could hinder the normal development of the cerebellum and IV ventricle [19]. Two theories have been proposed to explain the link between leptomeningeal melanosis and DWC. Chalpouka et al. [5] suggest that leptomeningeal melanosis restricts the ability of primitive meningeal cells from inducing normal deposition of the extracellular matrix, neuronal migration and formation of CSF resorption pathways, resulting in the formation of posterior fossa cysts and vermian aplasia characteristic of DWC. Barkovich et al. [4], on the other hand, have proposed that leptomeningeal melanosis interferes with normal ectodermal–mesodermal interaction, causing abnormal formation of the cerebellum and fourth ventricle.

## Diagnosis

Criteria for the diagnosis of NCM were first proposed in 1972 and included large or numerous pigmented nevi in patients without malignant transformation in cutaneous lesions and without evidence of melanoma except in the leptomeninges [12].

Since then, both malignant transformation of cutaneous nevi and distant metastases of leptomeningeal melanoma have been recorded [10]. This led to revision of the diagnostic criteria which currently comprise (1) large (diameter more than 20 cm in adults or 6–9 cm in infants) or numerous (three or more lesions) congenital nevi in association with leptomeningeal melanosis or melanoma; (2) no evidence of cutaneous melanoma, except in patients with histologically benign meningeal lesions; and (3) no evidence of meningeal melanoma, except in patients with histologically benign cutaneous lesions [19].

Confirmation of the diagnosis is still based on histological findings, often only at autopsy. Hairy nevi can also be found in up to 40% of cases of primary malignant melanoma of the leptomeninges [2]. In rare cases of leptomeningeal melanomatosis, tumour cells may be amelanotic (unpigmented) and patient may not present with NCM [36]. This highlights the difficulty of obtaining an histological diagnosis in these conditions.

On the other hand, MR imaging may allow a presumptive diagnosis of CNS melanosis to be made [13, 22]. Leptomeningeal melanosis demonstrates a distinctive hyperintensity on T1-weighted MR images and a hypointensity on T2-weighted MR images [4, 10]. Patients may also present with intraparenchymal lesions without meningeal involvement [32].

## Management

For cutaneous manifestations, the management remains controversial. Some dermatologists support prophylactic surgical excision of large melanocytic nevi to reduce the risk of malignant transformation, which occurs in 5 to 15% of patients [19], and to improve cosmetic appearance. For neurological manifestations, outcome remains poor even with the use of radiotherapy and chemotherapy [7, 23]. Early neurosurgical intervention, however, can assist in tissue diagnosis and has the potential to effect early decompression [13]. The usual surgical intervention is shunt insertion with a filter to prevent potential seeding into the abdominal space [29].

## Prognosis and outcomes

Prognosis in NCM is generally poor, with half of patients dying within 3 years of the onset of neurological symptoms [19]. However, the course of asymptomatic patients is variable and unpredictable [14, 20]. The worst prognosis is seen in NCM patients with Dandy–Walker complex (DWC). DWC is thought to be a marker of melanocytic infiltration into the CNS and confers an increased risk for malignant transformation [5].

## Exemplary case description

**Fig. 1:**
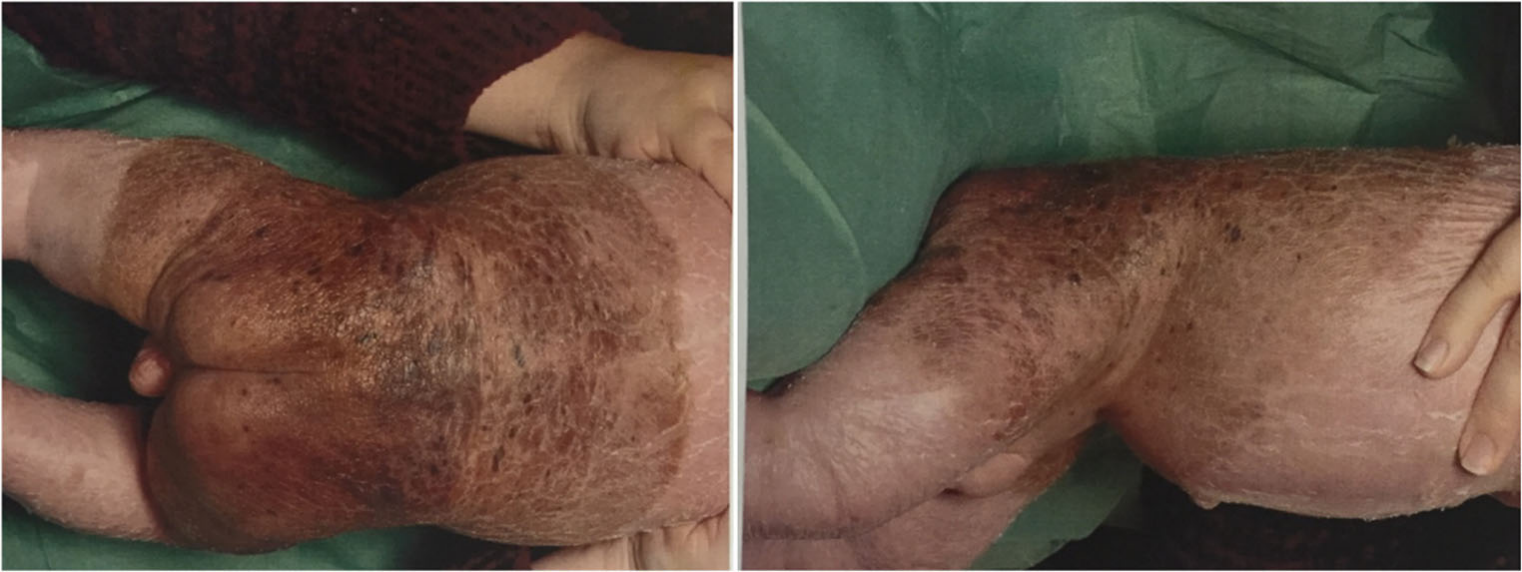
Giant hairy nevus in bathing trunk distribution

A 5-month-old male was delivered via caesarean section at 36-week gestational age. At birth, two extensive congenital hairy nevi had been observed; the first is a giant hairy nevus in bathing trunk distribution and the second 1 cm wide on the left upper back. Figure 1 illustrates the bathing trunk distribution of the giant nevus.

There were no issues with his feeding, weight gain or neurological development. At 5 months, he presented with a 5-day history of drowsiness, poor feeding, high pitched cry, nausea and vomiting.

**Fig. 2:**
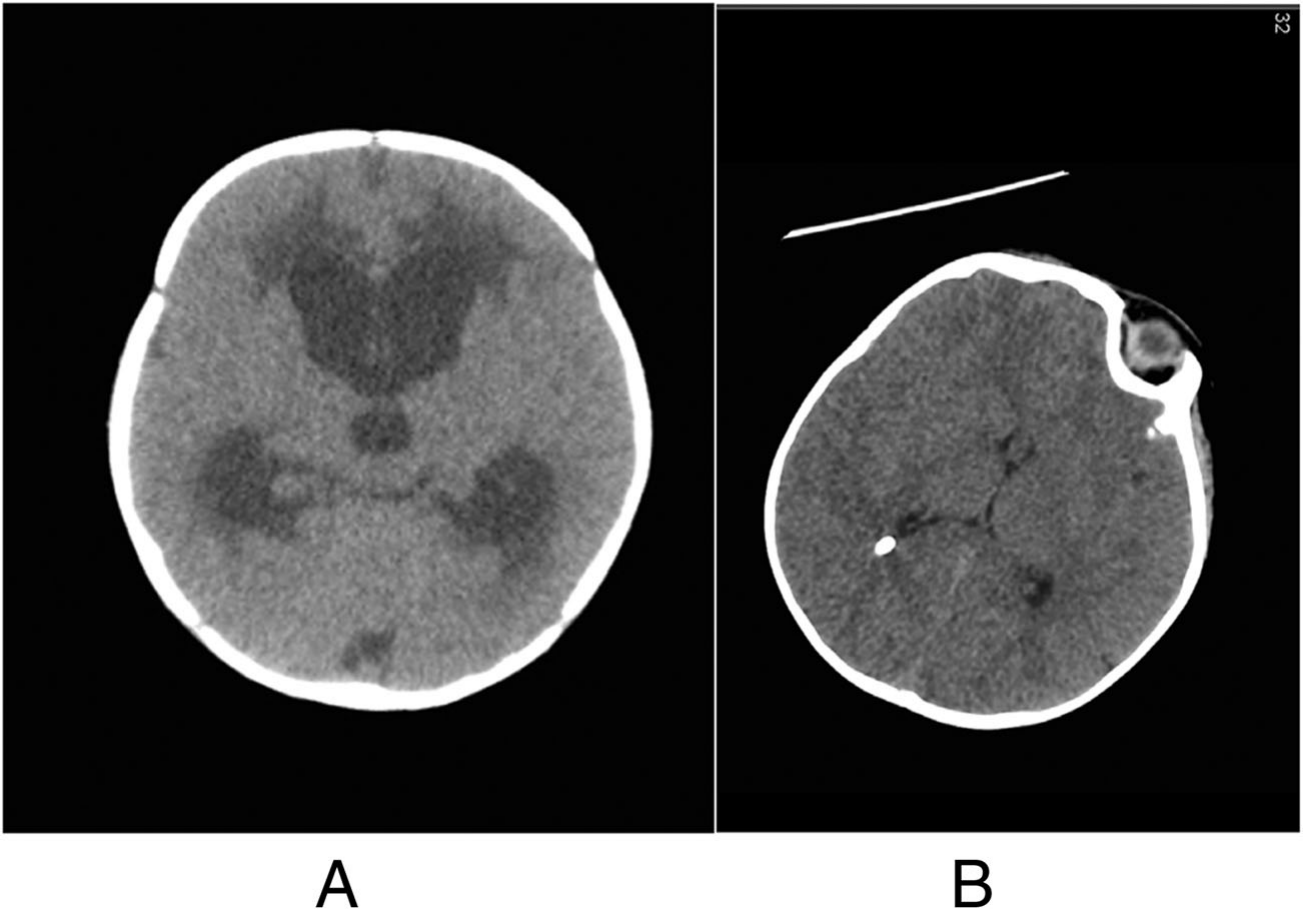
Axial CT. **a** Communicating hydrocephalous and transependymal oedema. **b** Decompressed ventricles after shunt placement

His skin was dry and flaky. His mother had mild psoriasis (elbows and knees) and benign moles, but there was no other relevant family history. At the time of presentation, he was alert but irritable, and his anterior fontanel was full and tense. Downward gaze (“setting-sun” sign) was also noticed intermittently but there was no neck rigidity. A non-enhanced computerised tomography (CT) scan demonstrated communicating hydrocephalus and significant transependymal oedema, as illustrated in Fig. 2.

**Fig. 3:**
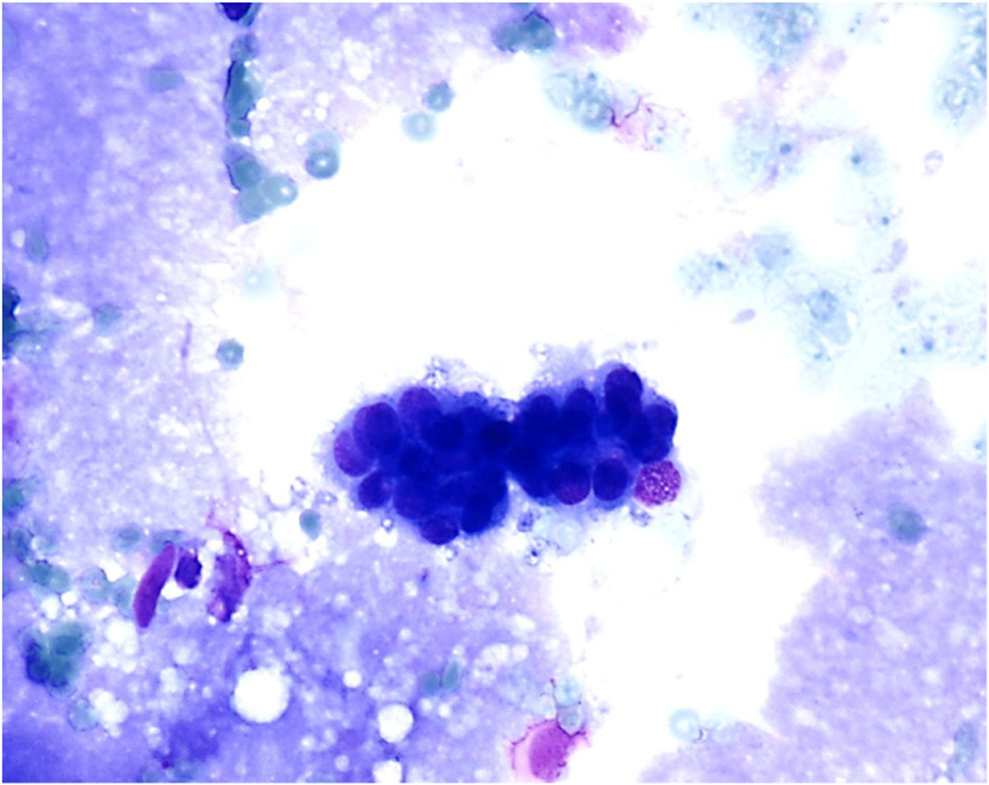
CSF cytology showing medium-sized epithelioid non-pigmented cells with oval nuclei and relatively high nuclear cytoplasmic ratios

An emergency right ventriculoperitoneal shunt was performed. The patient recovered well from the procedure and was discharged home on postoperative day one. The CSF was xanthochromic and cytological examination revealed medium-sized epithelioid non-pigmented cells with oval nuclei and relatively high nuclear cytoplasmic ratios. Such appearances have been described in children with NCM [22, 29]. Immunohistochemistry highlighted scattered lymphoid cells (CD45) and epithelioid cells were negative for melanoma markers (MelC, HMB45) as illustrated in Fig. 3.

**Fig. 4:**
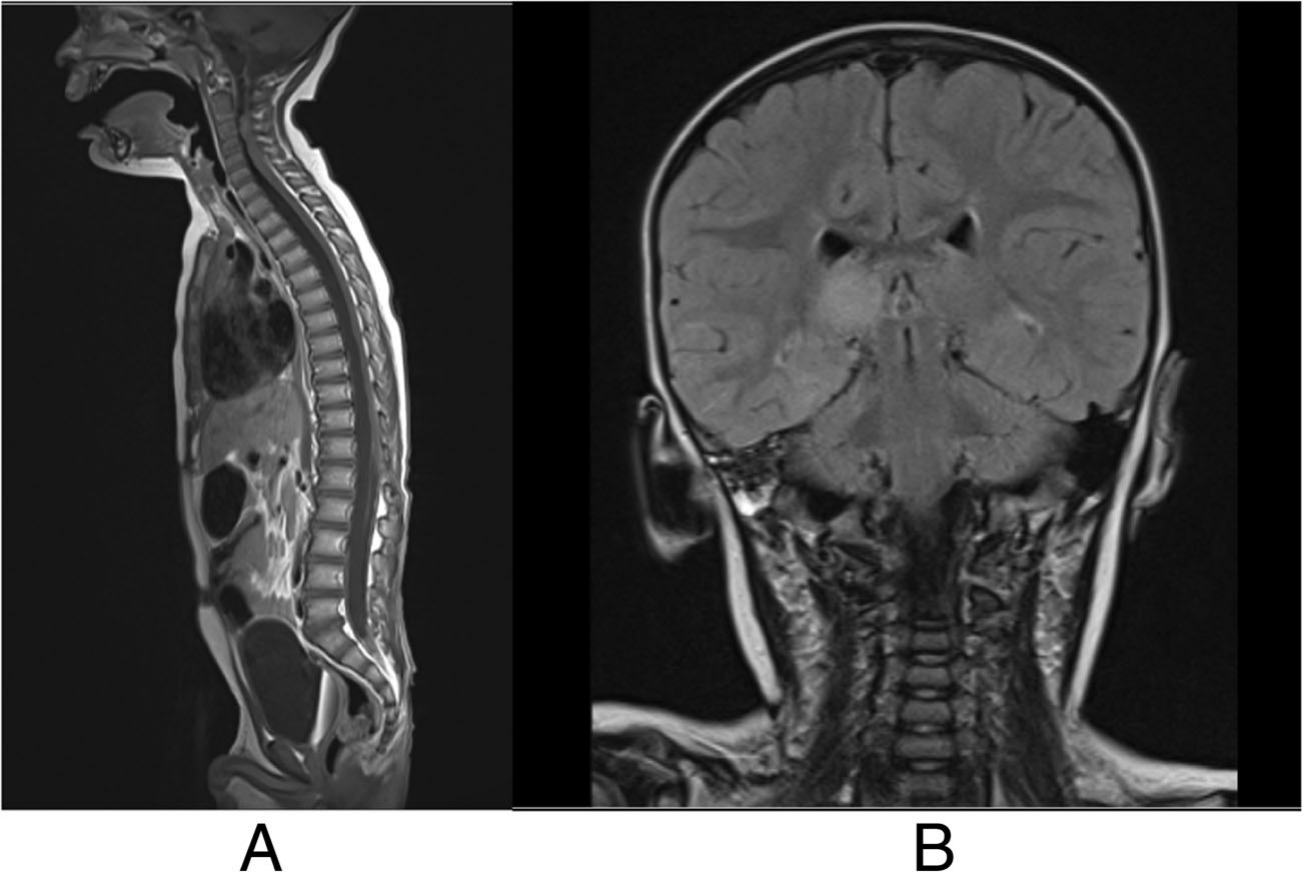
**a** T1-weighted MRI showing diffuse enhancement over the spinal cord again in keeping with CNS melanosis. **b** Coronal MRI flare image showing decompressed ventricles with oedema over the thalamus

MR imaging demonstrated meningeal enhancement in the periphery of the left and right cerebellum as well as in the thoracic spine and conus suggestive of melanin deposition. A presumptive diagnosis of NCM was made based on the MR characteristics, CSF cytology and clinical presentation. Follow-up MR 5 months after the procedure showed decompressed ventricles with oedema over the thalamus and diffuse enhancement over the spinal cord again in keeping with CNS melanosis, as illustrated in Fig. 4.

At 19 months of age, a repeat MRI showed arachnoid loculations at the ventricular outflow formina as well as thalamic oedema and diffuse spinal enhancement in keeping with NCM. Skin and leptomeningeal biopsies were subsequently taken at 21 months of age which showed N-type Rat Sarcoma gene (NRAS)-mutated melanoma, although PET scan showed no hypermetabolic foci within brain, spine or upper half of the body. For 7 months, he received trametinib, a MAPK/Erk kinase (MEK) inhibitor which inhibits cellular proliferation. During this time, he continued to develop normally and was attaining appropriate social and motor milestones. At 30 months of age, he developed left-sided weakness and status epilepticus requiring PICU admission and ventilator support. No acute changes were demonstrated on head and spine CT. He was extubated successfully but continued to deteriorate neurologically. He received palliative treatment and died at the age of 32 months.

## Conclusions

NCM is a rare syndrome characterised by congenital melanocytic nevi and melanocytic thickening of the leptomeninges. Cutaneous manifestations of NCM are usually congenital, and neurological manifestations develop early in life. Patients with large or multiple congenital nevi should therefore be investigated early—even in the absence of neurological manifestations—to facilitate treatment plan and prognosis. Because of the uncertain value of CSF cytology, MR imaging is the investigation of choice especially if biopsy cannot be carried out. As in the case reported here, symptomatic NCM usually presents with increased intracranial pressure and hydrocephalus and requires ventriculoperitoneal shunt insertion. Symptomatic NCM is refractory to radiotherapy and chemotherapy and has a poor prognosis. A multidisciplinary approach is necessary in the management of NCM patients. This should include routine neurodevelopmental assessments and dermatologist input.

## Abbreviations

NCM: Neurocutaneous melanosis
CNS: Central nervous system
HGF/SF: Hepatocyte growth factor/scatter factor
NRAS: N-type Rat Sarcoma gene
MAPK: Mitogen-activated protein kinase
MEK: MAPK/Erk kinase
FDA: The Food and Drug Administration
CSF: Cerebral spinal fluid
DWC: Dandy–Walker complex
PET: Positron emission tomography
PICU: Paediatric intensive care unit
DLE: Diffuse leptomeningeal enhancement
